# Experimentally-induced early recruitment of CD14+ monocytes in human allergic rhinitis

**DOI:** 10.1186/2045-7022-4-S2-O7

**Published:** 2014-03-17

**Authors:** Ibon Eguiluz-Gracia, Guro R Melum, Epsen S Baekkevold, Frode L Jahnsen

**Affiliations:** 1 Centre for Immune Regulation and Department of Pathology, Sognsvannsveien 20, Oslo, 0372, Norway; 2 Oslo University Hospital-Rikshospitalet, Centre for Immune Regulation and Department of Pat, Oslo, Norway

## Background

Allergic rhinitis (AR) is a complex inflammatory disorder where antigen presenting cells (APCs) are believed to activate allergen-specific CD4+ T cells at the site of allergen exposure. By sorting cells from the nasal mucosa in an experimental model of AR we have found, at the transcriptome level, that the total population of CD45+ HLA-DR+ cells showed signs of activation concomitant with an activated Th2 signature in purified CD4+ T cells. Bone marrow-derived APCs consists of many distinct subsets including dendritic cells (DC) and monocytes/macrophages. The aim of this work was to study the trafficking of various APC subsets during the allergic inflammation.

## Material and methods

Six AR-patients were challenged locally once a day with grass-pollen extract for 7 days out of the season. Daily symptoms were recorded using visual analogue scale. Mucosal biopsies were obtained before challenge and at day 3 and day 8. Multi-colour immunostaining *in situ* using a panel of antibodies was performed to determine the density of APC subsets. The density of eosinophils was also recorded.

## Results

All patients experienced typical symptoms throughout the challenge period and the density of tissue eosinophils was significantly increased at day 3 and 8 compared with baseline. At baseline HLA-DR+ cells could be clearly divided into two distinct subsets: the majority of cells were positive for the monocyte/macrophage marker CD14 whereas a minor population expressed the DC marker CD1c, but were negative for CD14. During the course of challenge a ~5-fold increase in the number of CD14+HLA-DR+ cells was observed at both day 3 and 7 (mean number of cells/mm2 76, 410 and 379 at baseline, day 3 and 7, respectively). The density of CD1c+ DCs, on the other hand, was not different from baseline to day 3, but showed a significant increase at day 7 (median number of cells/mm2 40 vs 120).

## Conclusion

We found a significant increase in the density of both CD14+ monocytes/macrophages and CD1c+DCs, but the increase of CD14+APCs appears earlier. CD14 is expressed on both macrophages and monocytes but in the setting of acute inflammatory reaction our findings strongly suggests that the increase is caused by an early recruitment of circulating CD14+ monocytes to the challenged site. This is in accordance with recent results in asthma mouse models lending further support to the notion that CD14+ "inflammatory" monocytes play a direct role in human airway allergy.

